# Immunotherapy in triple-negative breast cancer: mechanisms of resistance and emerging approaches: a narrative review

**DOI:** 10.12701/jyms.2026.43.17

**Published:** 2026-02-06

**Authors:** Sung Ae Koh

**Affiliations:** Division of Hemato-Oncology, Department of Internal Medicine, Yeungnam University College of Medicine, Daegu, Korea

**Keywords:** Immunotherapy, Review, Triple negative breast neoplasms

## Abstract

Triple-negative breast cancer (TNBC) is characterized by less treatment responsiveness and poorer prognosis than other breast cancer subtypes. The introduction of anti-programmed cell death 1 (PD-1)/programmed cell death ligand 1 (PD-L1) immunotherapy has expanded the therapeutic options beyond conventional chemotherapy, leading to the adoption of pembrolizumab-based regimens in both adjuvant and first-line palliative settings. However, in contrast to other tumor types that respond robustly to immune checkpoint inhibitors, the efficacy of PD-1/PD-L1 blockade in TNBC remains modest. Multiple factors contribute to this limited response, including the heterogeneity of PD-L1 expression, presence of an immunosuppressive tumor microenvironment regulated by complex immunomodulatory pathways, differences in mutational burden and neoantigen presentation, quantity and functional exhaustion of tumor-infiltrating lymphocytes, and variable synergy with combination partners. Numerous combination strategies have been actively investigated to enhance immunotherapeutic efficacy. Among these, antibody drug conjugates (ADCs) have shown the most promising results. The phase III ASCENT-04/KEYNOTE-D19 trial demonstrated that the combination of sacituzumab govitecan and pembrolizumab significantly improved progression-free survival in patients with PD-L1–positive metastatic TNBC, establishing this regimen as a potential new first-line standard, pending guideline adoption. Although the overall survival data are still immature, the trend appears to be favorable. Other ADCs are being explored in early phase studies, and targeted therapies such as poly(ADP-ribose) polymerase and protein kinase B inhibitors have also shown preliminary activity in smaller trials. Further refinement of these strategies through biomarker-driven, large-scale studies is warranted to identify the most effective combinations and to improve outcomes in patients with TNBC.

## Introduction

Breast cancer is the second most common cancer and ranks first in terms of cancer incidence among women in the United States [1]. In Korea, the incidence of other cancers is declining, while that of breast cancer is steadily increasing [2]. Breast cancer can be categorized by hormone positivity and human epidermal growth factor receptor 2 (HER2) positivity, and treatment methods depend on the classification, i.e., estrogen receptor-positive or progesterone receptor-positive breast cancer, HER2-positive breast cancer, or triple-negative breast cancer (TNBC), the latter of which is negative for all three markers. Among these, TNBC can only be treated with cytotoxic chemotherapy and not with hormonal drugs or targeted therapies, as these treatments are ineffective in TNBC [3].

Anti-programmed cell death 1 (PD-1)/programmed cell death ligand 1 (PD-L1) therapy, first demonstrated in non-small cell lung cancer (NSCLC) over a decade ago, has been shown to be effective in numerous cancers, either alone or in combination with cytotoxic chemotherapy or targeted agents [4]. It has since been widely used for the treatment of several cancers and is now essential for the treatment of NSCLC, renal cell carcinoma, malignant melanoma, and urothelial cell carcinoma [5-7]. In breast cancer, anti-PD-1/PD-L1 therapy has been actively researched as a treatment option for TNBC, which is resistant to hormonal therapy or HER2-targeted therapy. However, the results of studies on anti-PD-1/PD-L1 therapy have been disappointing. This article introduces the research on anti-PD-1/PD-L1 therapy for TNBC and discusses the limitations, underlying mechanisms of suboptimal efficacy, and strategies to overcome them.

## Studies of anti-programmed cell death therapy in triple-negative breast cancer

In 2019, IMpassion130, a phase III clinical trial conducted with nanoparticle albumin-bound (Nab)-paclitaxel with atezolizumab vs. placebo, demonstrated significantly improved progression-free survival (PFS), but not overall survival (OS) as a first-line treatment for metastatic TNBC (mTNBC). In a follow-up study, IMpassion131, PFS as the primary endpoint was not significantly improved; therefore, the approval of atezolizumab for mTNBC was voluntarily withdrawn by the pharmaceutical company in 2021. Based on the results of the KEYNOTE-522 trial, paclitaxel/carboplatin followed by doxorubicin/cyclophosphamide with pembrolizumab is currently used as preoperative chemotherapy for high-risk TNBC, regardless of PD-L1 expression (Table 1 [8-12]). In addition, pembrolizumab can be used as a first-line therapy with cytotoxic chemotherapy in cases with PD-L1 22C3 combined positive score (CPS)>10 based on the results of the KEYNOTE-355 trial in patients with mTNBC (Table 2 [13-18]). To the best of our knowledge, there are no additional reported data on anti-PD-1/PD-L1 therapy combined with cytotoxic chemotherapy for TNBC. Anti-programmed cell death (PD) therapy was expected to be a good option for patients with TNBC. However, disappointingly, these drugs do not show good efficacy in treating TNBC, unlike in other cancers. We discuss factors that are associated with these unfavorable results below.

## Factors that contribute to resistance to immunotherapy in triple-negative breast cancer

### 1. Heterogeneity in programmed cell death ligand 1 expression

PD-L1 expression correlates with positive responses to anti-PD-1/PD-L1 therapy in various cancer types [19]. TNBC is the most immunogenic breast cancer and has a higher PD-L1 expression than the other subtypes [20]. As mentioned above, pembrolizumab combined with chemotherapy was reported to be efficacious in patients with mTNBC and PD-L1 22C3 CPS >10 based on the KEYNOTE-355 trial [16]. mTNBC is characterized by an immunosuppressive tumor microenvironment (TME) in which pembrolizumab primarily reinvigorates preexisting exhausted T cells, rendering PD-L1 expression a critical predictive biomarker for clinical benefit. In contrast, in early-stage TNBC, a relatively preserved immune microenvironment and chemotherapy-induced antigen release enable pembrolizumab to induce de novo antitumor immunity, regardless of baseline PD-L1 expression [8]. Therefore, the association between PD-L1 positivity and the response to PD-1/PD-L1 therapy as a predictive biomarker should be discussed primarily in the context of mTNBC. Compared to melanoma or NSCLC, the overall PD-L1 expression levels in mTNBC are often lower or confined to the immune cell compartment rather than the tumor cells [21,22]. The location and pattern of PD-L1 expression may also influence drug efficacy.

In addition, there are some debates regarding the value of assessing PD-L1 positivity and companion diagnostic test results to identify patients for each anti-PD-1/PD-L1 therapy including pembrolizumab, nivolumab, atezolizumab, durvalumab, and avelumab. Some studies have indicated that SP263 and 22C3 assays for pembrolizumab tend to show increased PD-L1 staining compared with the SP142 assay for atezolizumab [23,24]. These results indicate that methods for testing PD-L1 expression level should be standardized and optimized.

### 2. Immunosuppressive tumor microenvironment

PD-L1 expression is reported to be modulated by multiple signaling pathways including the microRNA-200/ZEB1 axis, WNT, loss of PTEN, phosphoinositide 3-kinase (PI3K), and the MUC1-C/MYC/NF-κB axis, which might be related to multiple TME-related factors that can regulate the immune status of cancer cells in TNBC [25-28]. Refinement based on these various TME circumstances has classified TNBC into fully inflamed (FI), stroma-restricted, margin-restricted, and immune desert (ID) subtypes. The FI subtype, with rich immune cell infiltration, is particularly likely to benefit from PD-1/PD-L1 blockade [29,30]. Patients with TNBC and a higher proportion of stromal tumor-infiltrating lymphocytes (sTILs) responded well to pembrolizumab in the KEYNOTE-086 trial [31]. However, spatial and single-cell profiling revealed that many mTNBCs are the ID or immune-restricted subtypes, in which CD8⁺ T cells are scarce or sequestered at the stromal/tumor margins, respectively, creating chemokine and stromal barriers that predict attenuated responses to checkpoint blockade [32-34].

Even when present, tumor-infiltrating CD8⁺ T cells frequently exhibit exhaustion—sustained PD-1, TIM-3, and LAG-3 expression; reduced proliferation; impaired interferon gamma/interleukin 2/tumor necrosis factor alpha production; and diminished cytolysis—driven by chronic antigen exposure, metabolic stress, and suppressive cytokine signaling, and associated across solid tumors (including TNBC) with resistance to immune checkpoint inhibitors (ICIs) [35-39].

Compounding this, the TMEs of TNBC are often enriched in regulatory T lymphocytes (Tregs), myeloid-derived suppressor cells, and tumor-associated macrophages (M2 phenotype), creating a suppressive environment that hinders effector T cell function. Tregs are immunoregulatory CD4⁺ T cells that express the forkhead box P3 transcription factor. Tregs suppress excessive immune responses and prevent autoimmunity in normal tissues [40]. In the TME, Tregs are known to suppress antitumor immunity, induce the expression of PD-1, and help cancer cells evade immune responses [41].

Collectively, these spatial, cellular, and functional constraints explain why anti-PD-1/PD-L1 monotherapy often yields limited activity in mTNBC and underscore the rationale for combinatorial strategies that remodel the TME, enhance infiltration, and reinvigorate exhausted T cells.

### 3. Characteristics of mutational burden and neoantigens

Cancer-associated antigens arise from somatic mutations induced by external factors such as smoking and ultraviolet radiation. Some cancers (e.g., NSCLC, melanoma, and urothelial carcinoma) often exhibit a high tumor mutational burden (TMB) and generate numerous neoantigens, which in turn promote strong T cell responses [19]. TNBC lacks estrogen receptor/progesterone receptor/HER2 positivity and exhibits relatively higher genomic instability than other types of breast cancer but tends to have a lower persistent TMB and lower quality immunogenic neoantigens than melanoma and NSCLC [42,43]. As a result, CD8⁺ T cell priming is weak in TNBC, and the T cell repertoire capable of eliciting responses even after PD-1 blockade may be limited.

### 4. Interaction with combination therapies (chemotherapy, targeted agents)

While ICIs are used alone for melanoma and NSCLC, chemotherapy plus PD-L1 inhibitors (e.g., atezolizumab plus Nab-paclitaxel or pembrolizumab plus chemotherapy) is the standard treatment for PD-L1–positive TNBC [44-46]. Although anti-PD-1/PD-L1 therapy plus chemotherapy is currently widely used in patients with NSCLC, regardless of PD-L1 status, it can be used alone in patients with PD-L1–positive TNBC [47-50]. The reason for using chemotherapy with anti-PD-1/PD-L1 therapy combinations is that chemotherapy induces immunogenic cell death and remodels the TME, enhancing the efficacy of the PD-1/PD-L1 blockade [51,52]. This demonstrates that anti-PD-1/PD-L1 therapy alone is insufficient for the baseline immune state in TNBC. Even in mTNBC, combining anti-PD-1/PD-L1 antibodies with chemotherapeutic agents that show activity does not consistently improve outcomes. Although promising PFS results were observed with atezolizumab combined with Nab-paclitaxel in the IMpassion130 trial, this combination as a first-line therapy failed to show OS efficacy in patients with mTNBC [13]. In addition, the IMpassion131 study demonstrated that the combination of atezolizumab and paclitaxel did not improve PFS or OS in patients with mTNBC. Specifically, in the PD-L1–positive cohort, the median PFS was 6.0 months with the combination therapy compared to 5.7 months with paclitaxel alone (hazard ratio [HR], 0.82; *p*=0.20), and the median OS was 22.1 vs. 28.3 months (HR, 1.12), respectively [15]. In contrast, the KEYNOTE-355 trial demonstrated that pembrolizumab combined with various chemotherapeutic agents, including Nab-paclitaxel, paclitaxel, and gemcitabine plus carboplatin, significantly improved the PFS and OS in patients with PD-L1–positive mTNBC. In the PD-L1 CPS ≥10 subgroup, the median PFS was 9.7 months with the combination therapy compared to 5.6 months with chemotherapy alone (HR, 0.48; *p*=0.02), and the median OS was 23.0 vs. 16.1 months (HR, 0.54; *p*=0.04), respectively [17]. These findings suggest that the efficacy of atezolizumab in mTNBC is influenced by the choice of chemotherapy backbone, highlighting the need to identify optimal partner agents for combination therapy.

The current analyses suggest that the failure of atezolizumab treatment in mTNBC may be largely attributed to its restriction to taxane-based regimens. The major challenge in this field is the identification of the most effective partner agents to combine with anti-PD-1/PD-L1 therapy.

### 5. Molecular subtype and clonal heterogeneity

Although TNBC appears to be a single disease, it is comprised of a diverse mix of molecular subtypes. One study performed gene expression profiling of tumor samples from 587 patients with TNBC and divided the TNBC into six subtypes: basal-like 1, basal-like 2, mesenchymal, mesenchymal stem-like (MSL), immunomodulatory (IM), and luminal androgen receptor [53]. These subtypes differ in immune cell infiltration, TMB, and PD-L1 expression, which influences their responsiveness to anti-PD-1/PD-L1 therapy.

The IM subtype is characterized by abundant immune cell infiltration and high PD-L1 expression, rendering it more responsive to anti-PD-1/PD-L1 therapy. By contrast, the mesenchymal and MSL subtypes exhibit low immune cell infiltration and an immunosuppressive TME, which is associated with reduced responsiveness to anti-PD-1/PD-L1 therapy [53]. This high heterogeneity may limit the subtypes that benefit from PD-1 blockade and may explain the lower objective response rate (ORR) of anti-PD therapy across the entire patient population with TNBC, highlighting the importance of a personalized immunotherapeutic approach.

## Present status to overcome the challenges

Currently, many studies are attempting to find chemotherapy or targeted therapy as a counterpart to anti-PD-1/PD-L1 therapy to increase efficacy in the adjuvant or metastatic setting of TNBC. Recent clinical trials investigating PD-1/PD-L1 blockade-based combinations are summarized in Table 3 [54-67].

### 1. Antibody drug conjugates

Beyond chemotherapy, emerging strategies focus on combining PD-1/PD-L1 blockade with targeted agents. The first study to show visible results was the ASCENT-4/KEYNOTE-D19 phase III study with sacituzumab govitecan (SG) and pembrolizumab. SG is an antibody drug conjugate (ADC) that targets the TROP-2 protein, has been approved by the U.S. Food and Drug Administration for its effectiveness in TNBC when used alone, and is currently used to treat patients with mTNBC [68]. In the ASCENT-04/KEYNOTE-D19 phase III trial, the combination of SG and pembrolizumab was evaluated as a first-line treatment for patients with PD-L1–positive (CPS ≥10) mTNBC. A total of 443 patients were randomized in a 1:1 ratio to receive SG plus pembrolizumab or the investigator’s choice of chemotherapy (either gemcitabine/carboplatin or paclitaxel) plus pembrolizumab. Using a prespecified analysis (data cutoff: March 3, 2025), the study met its primary endpoint of improved PFS: 11.2 months (95% confidence interval [CI], 0.3–16.7) in the experimental arm vs. 7.8 months (95% CI, 7.3–9.3) in the control arm (HR, 0.65; 95% CI, 0.51–0.84; *p*<0.001), demonstrating a statistically significant and clinically meaningful benefit of the SG plus pembrolizumab combination. The ORR also favored the SG plus pembrolizumab arm (59.7% vs. 43.2%), with a complete response (CR) observed in 13% of patients compared to 8% in the chemotherapy arm. At the time of analysis, the OS data were not yet mature, with approximately 26% of expected events observed. A favorable trend toward improved OS was seen in the SG plus pembrolizumab group, with an HR of 0.89 (95% CI, 0.62–1.29), although statistical significance was not reached [54]. Therefore, SG with pembrolizumab may be a potential new standard first-line treatment in patients with PD-L1–positive mTNBC. Other ADCs, including ladiratuzumab vedotin, are currently being investigated in early phase trials (e.g., NCT03310957) [69].

### 2. Poly ADP-ribose polymerase inhibitors

Poly ADP-ribose polymerase (PARP) inhibitors represent another promising class of drugs, particularly for patients harboring breast cancer gene (*BRCA*) mutations or homologous recombination deficiency. Combinations of niraparib and pembrolizumab are being evaluated in multiple phase I/II studies (KEYNOTE-162) in BRCA-mutated TNBC, showing preliminary clinical activity and acceptable safety profiles [56]. Phase II/III studies have also been conducted on other PARP inhibitors, such as olaparib plus pembrolizumab, in patients with TNBC. For example, KEYLINK-009 did not meet its primary endpoint OS (olaparib plus pembrolizumab, 25.1 months vs. chemotherapy plus pembrolizumab, 23.4 months) and PFS (5.5 vs. 5.6 months) in all patients with TNBC (n=271), not even in patients with PD-L1 CPS ≥10 (n=130; PFS, 5.7 vs. 5.7 months). However, efficacy was demonstrated in patients with TNBC and mutated *BRCA*, showing longer median PFS (n=59, 12.4 vs. 8.4 months) [57]. Another olaparib study, the DORA trial (NCT03167619), evaluated olaparib with or without durvalumab as maintenance therapy in patients with platinum-sensitive mTNBC. Forty-five patients were randomized to receive either olaparib alone (n=23) or olaparib plus durvalumab (n=22). The study met its primary endpoint, showing improved PFS with both olaparib alone (median PFS, 4.0 months; 95% CI, 2.6–6.1) and olaparib plus durvalumab (median PFS, 6.1 months; 95% CI, 3.7–10.1) compared with the historical platinum-based control [58].

In the neoadjuvant setting, preliminary results from a window-of-opportunity phase I/II study (NCT03594396) evaluating short-course olaparib with single-dose durvalumab prior to standard neoadjuvant chemotherapy reported high pathological CR (pCR) rates. Among the 40 patients who underwent neoadjuvant therapy and surgery, 30 (75%) achieved pCR. These findings suggest that brief PARP inhibition combined with PD-L1 blockade augments chemosensitivity in triple-negative/estrogen receptor-low breast cancer, although peer-reviewed, full-text efficacy reports are not yet published [59].

### 3. Additional agents

Additional targeted strategies under investigation include PI3K/protein kinase B (AKT)-pathway inhibitors and anti-angiogenic agents combined with anti-PD-1/PD-L1 therapy. Early phase clinical studies combining PI3K/AKT-pathway inhibitors with ICIs for TNBC have demonstrated promising antitumor activities. In the MARIO-3 trial, eganelisib (a PI3Kγ inhibitor) with atezolizumab and Nab-paclitaxel achieved an ORR of approximately 55% and a disease control rate of 84%, regardless of PD-L1 expression [60]. Similarly, a phase Ib study of combinations of ipatasertib (an AKT inhibitor) with atezolizumab plus taxane as a first-line treatment for mTNBC produced ORRs of 44% to 63% and a median PFS of 5.4 to 7.4 months (NCT03800836) [61]. However, a phase III study (IPATunity130, NCT03337724) of combination ipatasertib with atezolizumab plus paclitaxel vs. placebo plus paclitaxel (168 vs. 87 patients, respectively) as a first-line treatment for mTNBC showed no statistically significant improvement in PFS (7.4 vs. 6.1 months; HR, 1.02; 95% CI, 0.71–1.45) and OS (24.4 vs. 24.9 months; HR, 1.08; 95% CI, 0.73–1.58) [62]. Currently, there is no definitive evidence supporting the clinical efficacy of AKT inhibitor-based combinations in TNBC, and predictive biomarkers of therapeutic benefits remain poorly defined.

Angiogenesis-targeted therapy combined with PD-1/PD-L1 blockade shows consistent activity in TNBC. In first-line treatment of unresectable locally advanced, mTNBC, the single-arm phase II ATRACTIB trial of atezolizumab+paclitaxel+bevacizumab met its primary endpoint with a median PFS of 11.0 months (95% CI, 9.0–13.4) and an ORR of 63% (CR, 14%), with benefit observed even in PD-L1-negative tumors (median PFS, 9.3 months; median OS, 24.5 months) [63]. As a later-line treatment, camrelizumab+apatinib (chemotherapy-free) yielded an ORR of 43.3% and a median PFS of 3.7 months in a phase II study, whereas triplet camrelizumab+apatinib+eribulin produced an ORR of 37% and a median PFS of 8.1 months in heavily pretreated mTNBC irrespective of PD-L1 status [64,65]. The LEAP-005 TNBC cohort (lenvatinib+pembrolizumab) showed an ORR of 32%, median PFS of 5.1 months, and median OS of 11.4 months after ≥1 prior line(s) of therapy [66]. In the neoadjuvant setting, the exploratory phase II NeoPanDa03 trial (camrelizumab+apatinib+chemotherapy) achieved a total pCR rate of 67.6% (23/34 patients) in patients with stage II–III TNBC, supporting further evaluation of vascular endothelial growth factor (VEGF) pathway modulation to enhance ICI efficacy [67]. Angiogenesis blockade (VEGF/VEGF receptor inhibitors) appears to reprogram the tumor–immune microenvironment by normalizing the vasculature, improving antigen/T cell trafficking, and dampening myeloid suppression, thereby potentiating anti-PD-1/PD-L1 therapy, with signals of activity even in PD-L1-negative TNBC. However, randomized phase III trials with prespecified composite biomarkers (angiogenesis/hypoxia signatures, TILs, and perfusion metrics) are needed to validate these benefits and optimize patient selection.

## Conclusion

Despite advances in immunotherapy, TNBC remains a therapeutically recalcitrant subtype of breast cancer. Although PD-1/PD-L1 blockade has broadened treatment options beyond chemotherapy, its clinical benefit remains limited compared to other cancers, reflecting the immunologic complexity of TNBC. Heterogeneous PD-L1 expression, an immunosuppressive TME, and exhausted T cell infiltration collectively dampen immune activation. Therefore, rational combinations are being explored to overcome these barriers. The phase III ASCENT-04/KEYNOTE-D19 trial demonstrated that SG plus pembrolizumab significantly improved PFS in patients with PD-L1–positive mTNBC, establishing a new benchmark for chemoimmunotherapy integration. Early data on PARP and AKT inhibitors have also indicated a potential synergy, warranting further validation. Ultimately, advances in TNBC immunotherapy will depend on refining combination partners and defining biomarkers that predict durable immune responsiveness.

## Figures and Tables

**Table 1.** Main studies with anti-PD-1/PD-L1 therapy in preoperative chemotherapy for early TNBC

Study name	KEYNOTE-522 [8,9]	NeoTRIPaPDL1 [10]	IMpassion031 [11]	GeparNUEVO [12] (phase II)
Population	T1c N1-2 or T2-4 N0-2 TNBC (n=1,174)	Early high-risk or locally advanced TNBC (n=280)	cT2-4 cN0-3 TNBC (n=333)	T1b-T4a-d TNBC (n=174)
Random	2:1 (784/390)	1:1 (138/142)	1:1 (165/168)	1:1 (88/86)
Anti-PD-1/PD-L1 therapy	Pembrolizumab	Atezolizumab	Atezolizumab	Durvalumab
Treatment	A: carboplatin and paclitaxel+pembrolizumab → AC/EC → surgery → pembrolizumab	A: carboplatin and nab-paclitaxel+atezolizumab → surgery → AC/EC	A: atezolizumab → nabpaclitaxel → AC	A: durvalumab+nabpaclitaxel → EC
	B: carboplatin and paclitaxel+placebo → AC/EC → surgery → placebo	B: carboplatin and nab-paclitaxel → surgery → AC/EC	B: placebo → nabpaclitaxel → AC	B: placebo+nabpaclitaxel → EC
Primary endpoint	pCR, defined as pathological stage ypT0/Tis ypN0 at the time of definitive surgery	pCR	pCR	pCR (ypT0/ypN0)
		IC testing positive (IC 1+, 2+, 3+) vs. no (IC 0)		Secondary endpoint:
				•Response by other pCR definitions (ypT0/Tis ypN0, ypT0/Tis ypN0/+, and ypN0)
				•Efficacy in predefined subgroups according to centrally assessed sTILs: low (≤10%), intermediate (11%–59%), high (≥60%)
PD-L1 definition	CPS ≥1	IC 0 (IC <1%)	IC ≥1	TC, IC ≥1% in one or both of these percentages
		IC 1+ (IC >1% and <5%)		
		IC 2+/3+ (IC ≥5%)		
PD-L1+ population	656 (83.7 %)	156 (IC 1+ 102; IC 2–3+ 54)	PD-L1 status (atezo/CTx-placebo/CTx	PD-L1 (durvalumab/placebo)
				Positive: 9/11
			Positive: 78 (47%), 76 (45%)	Negative: 69/69
			Negative: 87 (53%), 92(55%	Missing: 10
Assay	22C3 pharmDx assay	VENTANA SP142 assay		
pCR	260 vs. 103 (*p*<0.001)	ITT: 48.6% vs. 44.4%	ITT: 95 (57.6%) vs. 69 (41.1%), *p*=0.0044	ITT: 53.4% vs. 44.2%
	ITT: 63% vs. 55.6%	PD-L1 positive: 51.9% vs. 48% (no significant, *p*=0.48)		PD-L1 positive: 58% vs. 50.7%
	PD-L1 positive: 45.3% (29 of 64)	Multivariate analysis of pCR	PD-L1 positive: 53 (68.8%) vs. 37 (49.3%), *p*=0.021	Window-cohort: 61.0% vs. 41.4%
	PD-L1 negative: 30.3% (10 of 33)	Positive vs. negative 2.08 (1.64–2.65), *p*<0.0001		Not significant
EFS	3-year EFS: 84.5% vs. 76.8% (HR, 0.63; 95% CI, 0.48–0.82; *p*<0.001)	Not reported	Not reported	3-year iDFS: 84.9% vs. 76.9%
				3-year OS: 95.1% vs. 83.1% (EFS benefit)

PD-1, programmed cell death 1; PD-L1, programmed cell death ligand 1; TNBC, triple-negative breast cancer; AC, adriamycin cyclophosphamide; EC, epirubicin cyclophosphamide; pCR, pathological complete response; ypT0, post-treatment primary tumor stage 0; Tis, tumor in situ; ypN0, post-treatment nodal stage 0; IC, immune cells; CPS, combined positive score; sTILs, stromal tumor-infiltrating lymphocytes; TC, tumor cells; ITT, intention-to-treat; CTx, chemotherapy; HR, hazard ratio; CI, confidence interval; EFS, event-free survival; iDFS, invasive disease-free survival; OS, overall survival.

**Table 2.** Main studies with anti-PD-1/PD-L1 therapy in the first line for advanced TNBC

Study name	IMpassion130 [13,14] phase III	IMpassion 131 [15] phase III	Keynote-355 [16,17] phase III	TBCRC043 [18] phase II
Population	mTNBC (902)	mTNBC (651)	mTNBC (847)	130
Random	1:1	2:1	2:1	1:1
Anti-PD-1/PD-L1 therapy	Atezolizumab	Atezolizumab	Pembrolizumab	Atezolizumab
CTx	Nab-paclitaxel	Paclitaxel	Nab-paclitaxel	Atezolizumab/carboplatin 56
			Paclitaxel	Carboplatin 50
			Carboplatin/gemcitabine	
Primary endpoint	PFS and OS in ITT and PD-L1	PFS in PD-L1 positive and ITT (hierarchical)	PFS and OS in PD-L1 CPS score ≥10, ≥1, and ITT (hierarchical)	PFS
PD-L1 definition	IC >1	IC >1	CPS >1 and CPS >10	IC >1
PD-L1+ population	Total 368	Placebo/PAC (101)	CPS ≥1 (636)	IC >1: 22.2% (20 of 90)
	IC >1% and <5% (243)	Ate/PAC (191)	CPS ≥10 (323)	Most (90%) had PD-L1 positivity on stromal/immune cells, with only 2 specimens with PD-L1–positive tumor cells
	PD-L1 IC >5% (125)			
Assay	SP142	SP142	22C3	SP 142
PFS (mo)	ITT: 7.2 vs. 5.5 (HR, 0.80; 95% CI, 0.69–0.92; *p*=0.002)	ITT: 5.7 vs. 5.6 (HR, 0.86; 95% CI, 0.70–1.05)	ITT: 7.5 vs. 5.6 (HR, 0.82; 95% CI, 0.70–0.98)	Median PFS
	PD-L1: 7.5 vs. 5.3 (HR, 0.63; 95% CI, 0.50–0.80)	PD-L1: 6.0 vs. 5.7 (HR, 0.82; 95% CI, 0.60–1.12; *p*=0.20)	PD-L1 CPS ≥10: 9.7 vs. 5.6 (HR, 0.66; 95% CI, 0.50–0.88)	4.1 vs. 2.2 (HR, 0.66; *p*=0.05)
			PD-L1 CPS ≥1: 7.5 vs. 5.6 (HR, 0.75; 95% CI, 0.62–0.91)	Regardless of PD-L1 status
OS (mo)	ITT: 21.0 vs. 18.7 (HR 0.87; 95% CI, 0.75–1.02; *p*=0.08)	ITT: 19.2 vs. 22.8 (HR, 1.12; 95% CI, 0.88–1.43)	ITT: 17.2 vs. 15.5 (HR, 0.89; 95%CI, 0.76–1.05)	Median OS
	PD-L1: 25.4 vs. 17.9 (HR, 0.67; 95% CI, 0.53–0.86)	PD-L1: 22.1 vs. 28.3 (HR, 1.11; 95% CI, 0.76–1.64)	PD-L1 CPS ≥10: 23.0 vs. 16.1 (HR, 0.73; 95% CI, 0.55–0.95; *p*=0.0185)	12.6 vs. 8.6 (HR, 0.60; *p*=0.03)
			PD-L1 CPS ≥1: 17.6 vs. 16.0 (HR, 0.86; 95% CI, 0.72–1.04, *p*=0.1125)	

PD-1, programmed cell death 1; PD-L1, programmed cell death ligand 1; TNBC, triple-negative breast cancer; mTNBC, metastatic triple-negative breast cancer; CTx, chemotherapy; PFS, progression-free survival; OS, overall survival; ITT, intention-to-treat; IC, immune cells; CPS, combined positive score; HR, hazard ratio; CI, confidence interval.

**Table 3.** Recent studies of PD-1/PD-L1 combinations with chemotherapy/targeted agents

Category	Study/ Trial ID	Phase	Setting	Population/eligibility	Arm/backbone	Number	Primary endpoint/result (mo)	ORR	PFS (mo)	OS (mo)	Note
ADC+PD-1	ASCENT-04/KEYNOTE-D19 [54]	Phase III	1L mTNBC, PD-L1 CPS ≥10	Untreated mTNBC; PD-L1–positive	Sacituzumab govitecan+pembrolizumab vs. chemo (gem/carb or taxane)+pembrolizumab	443 total (1:1)	PFS 11.2 vs. 7.8; HR 0.65; *p*<0.001	59.7% vs. 43.2% (CR 13% vs. 8%)	11.2 vs. 7.8	Immature (HR 0.89)	Potential new 1L standard in PD-L1+ mTNBC
ADC+PD-1	NCT03310957 [55]	Phase Ib/II (ongoing)	1L mTNBC	Unresectable locally advanced or mTNBC	Ladiratuzumab vedotin+pembrolizumab	NA	Early-phase signal; ongoing	NA	NA	NA	Early-phase investigation
PARP+PD-1	KEYNOTE-162 [56]	Phase I/II	Pre-treated TNBC	BRCA-mutated TNBC (per user summary)	Niraparib+pembrolizumab	NA	Preliminary clinical activity; acceptable safety	NA	NA	NA	Preliminary signal
PARP+PD-1	KEYLYNK-009 [57]	Phase II/III	Post-induction mTNBC	All-comers; PD-L1 CPS ≥10 subgroup; BRCA-mut subgroup	Olaparib+pembrolizumab vs. chemo+pembrolizumab	271 total; PD-L1 CPS ≥10 n=130; BRCA-mut n=59	OS (25.1 vs. 23.4) PFS (5.5 vs. 5.6) (NS)	NA	5.5 vs. 5.6 (overall); BRCA-mut 12.4 vs. 8.4	25.1 vs. 23.4 (NS)	Benefit signal in BRCA-mut subgroup
PARP+PD-L1	DORA (NCT03167619) [58]	Phase II	Maintenance in platinum-sensitive mTNBC	Responders (CR/PR/SD) to prior platinum	Olaparib vs. olaparib+durvalumab	22 vs. 23	Both arms improved vs. historical platinum continuation	NA	6.1 (combo) vs. 4.0 (mono)	NA	Chemo-free maintenance strategy
PARP+PD-L1	NCT03594396 [59]	Phase I/II	Neoadjuvant (pre-NACT)	TNBC/ER-low; stage II–III	Short course olaparib+one dose durvalumab → standard NACT	40 evaluable for surgery	pCR 75% (30/40) after NACT	NA	NA	NA	Preliminary; peer-reviewed full text pending
PI3Kγ+PD-L1	MARIO-3 [60]	Phase II (single-arm)	1L mTNBC	All-comers, PD-L1-agnostic	Eganelisib+atezolizumab+nab-paclitaxel	NA	ORR ~55%; DCR 84%	~55%	NA	NA	Signal regardless of PD-L1
AKT+PD-L1	NCT03800836 [61]	Phase Ib	1L mTNBC	NA	Ipatasertib+atezolizumab+paclitaxel/nab-paclitaxel	NA	ORR 44%–63%; mPFS 5.4–7.4	44%–63%	5.4–7.4	NA	
AKT+PD-L1	IPATunity130 (NCT03337724) [62]	Phase III	1L mTNBC	NA	Ipatasertib+atezolizumab+paclitaxel vs. placebo+paclitaxel	168 vs. 87	Negative: PFS 7.4 vs. 6.1 (HR 1.02); OS 24.4 vs. 24.9 (HR 1.08)	NA	7.4 vs. 6.1 (NS)	24.4 vs. 24.9 (NS)	No significant improvement
Anti-angiogenic+PD-L1	ATRACTIB [63]	Phase II (single-arm)	1L locally advanced metastatic TNBC	All-comers; PD-L1–unselected	Atezolizumab+paclitaxel+bevacizumab	NA	Primary met: mPFS 11.0; ORR 63% (CR 14%)	63% (CR 14%)	11.0 (PD-L1–neg: 9.3)	24.5 in PD-L1–neg (descriptive)	Signal in PD-L1–negative tumors
Anti-angiogenic+PD-1	NCT03394287 [64]	Phase II (single-arm)	Later-line mTNBC	Pre-treated	Camrelizumab+apatinib	NA	ORR 43.3%; mPFS 3.7	43.3%	3.7	NA	Chemo-free doublet
Anti-angiogenic+PD-1+chemo	NCT04303741 [65]	Phase II (single-arm)	Later-line mTNBC	Heavily pretreated; PD-L1–irrespective	Camrelizumab+apatinib+eribulin	NA	ORR 37%; mPFS 8.1; DCR 87%	37%	8.1	NA	Manageable toxicity
Anti-angiogenic+PD-1	LEAP-005 TNBC cohort [66]	Phase II (single-arm)	Post-line mTNBC	≥1 prior lines	Lenvatinib+pembrolizumab	NA	ORR 32%; mPFS 5.1; mOS 11.4	32%	5.1	11.4	Manageable safety
Anti-angiogenic+PD-1+chemo	NeoPanDa03 [67]	Phase II (exploratory)	Neoadjuvant (stage II–III)	TNBC	Camrelizumab+apatinib+chemotherapy	34 (tpCR evaluable)	tpCR 67.6% (23/34)	NA	NA	NA	Supports VEGF-pathway modulation in neo-adjuvant setting

PD-1, programmed cell death 1; PD-L1, programmed cell death ligand 1; ADC, antibody drug conjugate; PARP, poly ADP-ribose polymerase; PI3Kγ, phosphoinositide 3-kinase gamma; AKT, protein kinase B; ORR, objective response rate; PFS, progression-free survival; OS, overall survival; HR, hazard ratio; CR, complete response; NA, not applicable; 1L, first line; mTNBC, metastatic triple-negative breast cancer; CPS, combined positive score; BRCA, breast cancer gene; NS, not significant; DCR, disease control rate; mPFS, median progression-free survival; mOS, median overall survival; ER, estrogen receptor; NACT, neoadjuvant chemotherapy; pCR, pathological complete response; tpCR, total pathological complete response; VEGF, vascular endothelial growth factor.
